# Chronic–Progressive Dopaminergic Deficiency Does Not Induce Midbrain Neurogenesis

**DOI:** 10.3390/cells10040775

**Published:** 2021-03-31

**Authors:** Mareike Fauser, Francisco Pan-Montojo, Christian Richter, Philipp J. Kahle, Sigrid C. Schwarz, Johannes Schwarz, Alexander Storch, Andreas Hermann

**Affiliations:** 1 Department of Neurology, University Medical Center Rostock, 18147 Rostock, Germany; mareike.fauser@med.uni-rostock.de (M.F.); alexander.storch@med.uni-rostock.de (A.S.); 2 Department of Neurology, Technische Universität Dresden, 01307 Dresden, Germany; info@augenarzt-waldkirch.de; 3 Munich Cluster for Systems Neurology, Department of Psychiatry, University Hospital LMU, 80336 Munich, Germany; Francisco.Pan-Montojo@med.uni-muenchen.de; 4 Laboratory of Functional Neurogenetics, Department of Neurodegeneration, Hertie Institute for Clinical Brain Research, 72076 Tübingen, Germany; philipp.kahle@uni-tuebingen.de; 5 German Centre for Neurodegenerative Diseases (DZNE), 72076 Tübingen, Germany; 6 Department of Neurology, University Hospital Leipzig, 04103 Leipzig, Germany; s.schwarz@curiositas-ad-sanum.de (S.C.S.); Johannes.Schwarz@innklinikum.de (J.S.); 7 Department of Neurology, Klinik Haag i. OB, 83527 Oberbayern, Germany; 8 German Centre for Neurodegenerative Diseases (DZNE) Rostock-Greifswald, 18147 Rostock, Germany; 9 Center for Transdisciplinary Neurosciences Rostock (CTNR), University Medical Center Rostock, University of Rostock, 18147 Rostock, Germany; 10 Translational Neurodegeneration Section “Albrecht Kossel”, Department of Neurology, University Medical Center Rostock, 18147 Rostock, Germany

**Keywords:** adult neurogenesis, periventricular regions, non-neurogenic regions, Parkinson´s disease, dopaminergic neurodegeneration, transgenic animal model

## Abstract

Background: Consecutive adult neurogenesis is a well-known phenomenon in the ventricular–subventricular zone of the lateral wall of the lateral ventricles (V–SVZ) and has been controversially discussed in so-called “non-neurogenic” brain areas such as the periventricular regions (PVRs) of the aqueduct and the fourth ventricle. Dopamine is a known modulator of adult neural stem cell (aNSC) proliferation and dopaminergic neurogenesis in the olfactory bulb, though a possible interplay between local dopaminergic neurodegeneration and induction of aNSC proliferation in mid/hindbrain PVRs is currently enigmatic. Objective/Hypothesis: To analyze the influence of chronic–progressive dopaminergic neurodegeneration on both consecutive adult neurogenesis in the PVRs of the V–SVZ and mid/hindbrain aNSCs in two mechanistically different transgenic animal models of Parkinson´s disease (PD). Methods: We used Thy1-m[A30P]h α synuclein mice and Leu9′Ser hypersensitive α4* nAChR mice to assess the influence of midbrain dopaminergic neuronal loss on neurogenic activity in the PVRs of the V–SVZ, the aqueduct and the fourth ventricle. Results: In both animal models, overall proliferative activity in the V–SVZ was not altered, though the proportion of B2/activated B1 cells on all proliferating cells was reduced in the V–SVZ in Leu9′Ser hypersensitive α4* nAChR mice. Putative aNSCs in the mid/hindbrain PVRs are known to be quiescent in vivo in healthy controls, and dopaminergic deficiency did not induce proliferative activity in these regions in both disease models. Conclusions: Our data do not support an activation of endogenous aNSCs in mid/hindbrain PVRs after local dopaminergic neurodegeneration. Spontaneous endogenous regeneration of dopaminergic cell loss through resident aNSCs is therefore unlikely.

## 1. Introduction

With increasing life expectancy, neurodegenerative diseases such as Parkinson´s disease (PD) or Alzheimer´s disease affect a growing number of individuals [1]. Despite extensive clinical and preclinical research efforts, available treatment options are still exclusively symptomatic in nature with persistent disease progression [2,3]. Since adult neurogenesis is a well-described phenomenon in distinct brain regions, such as the ventricular–subventricular zone (V–SVZ) of the lateral wall of the lateral ventricles [4], the dentate gyrus (DG) of the hippocampus [5] and the hypothalamus along the third ventricle [6,7], endogenous regeneration as a therapeutic tool to reduce the impact of dopaminergic deficits in PD has been discussed for quite some time [8,9]. The neurogenic cascade in the V–SVZ has already been described in detail [10]; GFAP^+^ astrocytes (type B cells) located close to the ventricular lumen act as residing neural stem cells with comparatively low proliferative activity and generate rapidly-proliferating nestin^+^ type C cells. Type B cells can be further divided into B1 and B2 cells, with type B1 cells identified as the actual aNSCs. Activated B1 cells express nestin, while they are nestin^−^ in their quiescent state. Nestin^+^ type A cells or neuroblasts are the downstream progeny of type C cells and migrate along the rostral migratory system into the olfactory bulb (OB) to differentiate in GABAergic and dopaminergic neurons [11]. Dopamine acts via D2-like receptor activation to regulate V–SVZ precursor cell proliferation, and dopamine depletion reduces both proliferation in the V–SVZ and numbers of new neurons in the OB in animal models of acute dopaminergic deficiency [12,13], though human studies are so far limited in numbers and have revealed conflicting results [13,14].

Regarding a putative endogenous dopaminergic rescue, the presence or absence of neurogenic activity in the adult midbrain has long been a controversial issue. While initial studies postulated a constant turnover of dopaminergic cells in the substantia nigra even under physiological conditions with stimulation of proliferation after dopaminergic cell loss in vivo [15], later reports were not able to reproduce these findings; there was either no detectable proliferation in midbrain areas outlying the periventricular regions (PVRs) [16,17,18] or a lack of functional dopaminergic neurons, either due to retention of an undifferentiated state or due to mere gliogenesis [15,18,19,20,21,22,23,24,25].

These initial studies have been conducted in both healthy rodents and in established toxin-induced models, namely the 1-methyl-4-phenyl-1,2,3,6-tetrahydropyridine (MPTP) and 6-hydroxydopamine (6-OHDA) models [26,27]. However, these toxin-based models can only in part mimic the human disease course, e.g., concerning their lack of α synuclein-containing inclusion bodies (Lewy bodies) or the absence of a chronic-progressive disease manifestation, but do provide the opportunity to study different degrees of dopaminergic deficiency depending on the site and type of lesion (reviewed in [28]). In recent years, novel genetic PD animal models became available, which present, for example, with overexpression of mutated human (A30P) (m(A30P) h) α-synuclein under different promotors [29,30]. Another possible mechanism of progressive nigral dopaminergic cell loss is seen in Leu9′Ser α4* nAChR mice, in which chronic activation of nicotinergic receptors due to a point mutation in the α4 receptor subunit leads to a modest dopaminergic neuron degeneration during adulthood, though this model is only viable with reduced receptor expression. However, a strain with an inducible increase in receptor expression during adulthood is also available which displays a significant nigral loss of up to 70% of dopaminergic neurons and more pronounced motor deficits [31,32,33]. Other genetic models, such as the Pitx3-mutant mouse, are less suitable since they already present with a very early, even embryonic, dopaminergic cell loss [34].

Although we and others have already demonstrated the presence of quiescent adult neural stem or progenitor cells (aNSCs) in the periventricular regions of the adult mouse brain in vivo [16,35,36] with dopaminergic differentiation potential in vitro [15,37], the present study investigates the putative activation of such quiescent aNSCs through local neurodegenerative cues in two different transgenic PD models, namely the m[A30P]h α-synuclein mouse expressing the mutated human protein under the Thy1 promotor to ensure for high neuronal expression and the Leu9′Ser hypersensitive α4* nAChR mouse as described above [29,33]. Though the authors are well aware of the fact that mid/hindbrain neural stem or progenitor cells might not fulfill the strict criteria of stemness and might be of a more restricted progenitor cell type, we will adhere to the term “neural stem cells (NSCs)” throughout the paper to facilitate reading.

## 2. Materials and Methods

### 2.1. Animals

We used two different animal models of Parkinson´s disease. The Thy1-m[A30P]h α-synuclein mouse expresses mutant human [A30P]α-synuclein under the Thy1 promoter to achieve stable neuronal expression throughout the brain [38,39]. The Leu9′Ser α4* nAChR mouse is a heterozygous knock-in model with a hypersensitive nicotinic α4-receptor subunit, which presents with an early degeneration of nigral dopaminergic neurons mediated through cholinergic excitotoxicity as described earlier [31,32,33]. Both transgenic animal strains were generated on a C57BL/6 background, which was also used for controls (Charles River Laboratories, Sulzfeld, Germany). All animals were kept under standard laboratory conditions (12:12 h light–dark cycle, constant temperature and humidity and ad libitum access to food and water).

All animal experiments were conducted at the Department of Neurology, Leipzig University Hospital, Germany approved by local authorities (Landesdirektion Sachsen, Chemnitz, Germany). Ten to twelve week-old Thy1-m[A30P]h mutant mice, 16 ± 2 weeks old Leu9´Ser hypersensitive α4* nAChR receptor mice or age-matched C57BL/6 mice were intraperitoneally injected with 50 mg/kg bodyweight bromodeoxyuridine (BrdU; Sigma-Aldrich, St Louis, MO, USA) once daily for three consecutive days, anaesthetized with 50 mg/kg phenobarbital and intracardially perfused with 4% paraformaldehyde (PFA) the day after the last BrdU injection. Brains were extracted in toto and kept in 4% PFA for 24 h followed by overnight immersion in 15% and 30% sucrose, respectively. Brains were snapfrozen in dry ice powder and kept at −80 °C until further processing.

### 2.2. Immunohistochemistry

For quadruple immunofluorescence stainings, 25 μm frozen coronal sections from regions of interest were processed as described earlier [16]. In brief, free-floating cryosections were preincubated in 3% blocking serum containing 0.2% Triton X-100 in PBS for 2 h at room temperature, then primary antibodies were added overnight at 4 °C followed by secondary fluorescence-conjugated antibodies for 1 h at room temperature. For the detection of BrdU incorporation, sections were then incubated for 30 min in 1.5 N HCl at 37 °C and treated as described earlier. Cell nuclei were counterstained with 4,6-diamidino-2-phenylindole (DAPI). Primary antibodies were as follows: mouse anti-nestin monoclonal antibody (1:500; Chemicon International, California, CA, USA), rat anti-BrdU monoclonal antibody [BU1/75 (ICR1)] (1:150–1:200; Abcam, GB), rabbit anti-glial fibrillary acidic protein (GFAP) polyclonal antibody (1:1000–1:1500; Chemicon International), rabbit anti-tyrosine hydroxylase polyclonal antibody (TH; 1:1000; Pel-Freez, California, CA, USA), rat anti dopamine transporter monoclonal antibody (DAT; 1:5000; Millipore, California, CA, USA). Respective secondary antibodies were as follows: Alexa Fluor 488-conjugated donkey anti-mouse IgG, Alexa Fluor 594-conjugated donkey anti-rat IgG, Alexa Fluor 647-conjugated donkey anti-rabbit IgG (all 1:500; all from Molecular Probes, Eugene, OR, USA).

### 2.3. Quantitative Histology and Statistics

The coordinates of the coronal sections of interest were 0.3 to 0.1 mm for the lateral ventricles (LV), −4.2 to −4.4 mm for the aqueduct (Aq) and −5.4 to −5.6 mm for the fourth ventricle (4V; all from bregma). For quantification of the numbers of cells expressing a given marker/marker combination, numbers of positive cells were determined relative to total numbers of DAPI-labelled nuclei or BrdU-labelled proliferating cells, respectively, using a Leica DMIRE 2 laser scanning confocal microscope (Leica Microsystems, Wetzlar Germany) with Leica LAS AF Lite Software (version 2.0). For quantitative analyses, sections from three to four different animals per group were used (see figure captions for details), and each region was quantified in at least five separately stained coronal sections (see above for regional coordinates). To avoid counting biases, all cells (DAPI-stained nuclei) within the first 300 μm of the lateral wall ventral of the beginning RMS were counted for the LV, the complete circumference of the Aq and the median 300 μm of the ventral wall of the 4V. The following marker combinations were used: GFAP^+^/nestin^−^ for quiescent B1 cells, GFAP^+^/nestin^+^ for B2/activated B1 cells and GFAP^−^/nestin^+^ for downstream neuroprogenitor cells (type A and type C cells). Although absolute cell counts were theoretically possible with this procedure, comparisons of absolute cell counts do not seem useful because of the very different structure of the PVRs of the investigated regions and only relative cell counts normalized to either total cell counts (DAPI^+^ nuclei) or total BrdU^+^ cells are presented. Three-dimensional overlay was used to avoid false-positive/negative overlay and double counting. For quantification of striatal dopaminergic innervation by means of tyrosine hydroxylase (TH) and dopamine transporter (DAT) fluorescence intensity measurements, the mean fluorescence intensity in the striatum was normalized to the mean fluorescence intensity in non-striatal regions of the brain (signal-to-background ratio) as described earlier [32]. Statistical comparisons were made with either Student´s *t*-test or Mann–Whitney-U test, as appropriate, with SPSS Software 27.0 (IBM, New York, NY, USA). All data are presented as mean ± standard error of the mean (S.E.M.) from at least three individual animals.

## 3. Results

### 3.1. Degree of Dopaminergic Deficiency

The extent of dopaminergic deficiency in our transgenic animal strains is strongly age-dependent due to the chronic–progressive nature of neurodegeneration; it has already been demonstrated that Leu9′Ser α4* nAChR mice present with a reduction of both nigral dopaminergic cell loss and striatal TH^+^ dopaminergic nerve terminals of ~30–40% [32]. In the Thy1-m[A30P]h α-synuclein mice from this study, we found a reduction in TH^+^ dopaminergic terminals of ~50% and a reduction in dopamine transporter (DAT) intensity of ~30% (DAT_m[A30P]h_: 2.29 ± 0.27 (S.E.M.), TH_m[A30P]h_: 1.94 ± 0.26, *n* = 4; DAT_WT_: 3.38 ± 0.26; TH_WT_: 3.98 ± 0.13; *n* = 4).

### 3.2. Minor Alterations in V–SVZ Neurogenesis

**Figure 1:**
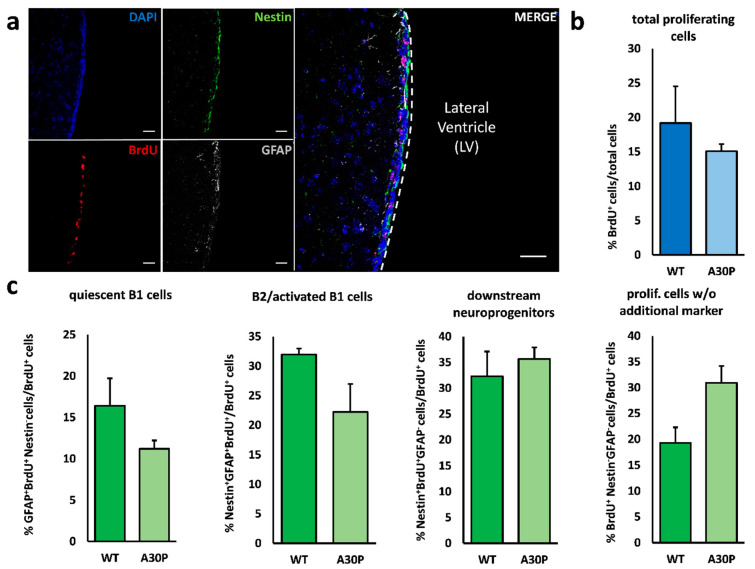
Neurogenesis in the V–SVZ of the Thy1-m[A30P]h α-synuclein mouse. (**a**) Representative fluorescence images of the ventricular–subventricular zone lining the lateral ventricle. Daily BrdU injections over 3 days labelled proliferating cells (red). To differentiate between the various cell types within the V–SVZ, quadruple immunostainings revealed GFAP^+^/nestin^−^ (grey) quiescent type B1 cells, nestin^+^/GFAP^−^ (green) type A and C cells (downstream neuroprogenitors) and GFAP^+^/nestin^+^ B2/activated B1 cells. Cell nuclei were counterstained with DAPI (blue). (**b**) Quantitative immunohistochemistry revealed no difference in the total number of proliferating, i.e., BrdU-incorporating, cells between wild-type and m[A30P]h α-synuclein mice. (**c**) In addition, we analyzed the relative distribution of the proliferating cell types in the V–SVZ but found no significant differences between m[A30P]h α synuclein animals and controls, though a tendency towards a reduction in the relative amount of B2/activated B1 cells was noticed. The proportion of quiescent type B1 cells and downstream neuroprogenitor cells (type A and C cells) was unchanged. Scale bar = 50 µm. All data are presented as mean ± standard error of the mean (S.E.M.); *n* = 3 each Abbreviations: LV—lateral ventricle; WT—wild-type; m[A30P]h—mutant human A30P α-synuclein mice; BrdU—bromodeoxyuridine; GFAP—glial fibrillary acidic protein; V–SVZ—ventricular–subventricular zone.

**Figure 2:**
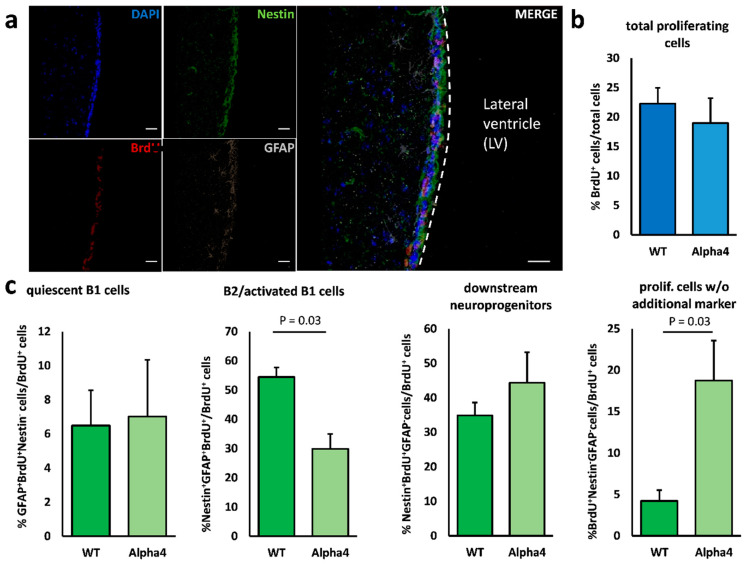
Neurogenesis in the V–SVZ of the L’S hypersensitive α4* nAChR mouse. (**a**) Representative fluorescence image of the ventricular–subventricular zone lining the lateral ventricle. Daily BrdU injections over 3 days labelled proliferating cells (red). To differentiate between the various cell types within the V–SVZ, quadruple immunostainings revealed GFAP^+^/nestin^−^ (grey) quiescent type B1 cells, nestin^+^/GFAP^−^ (green) type A and C cells (downstream neuroprogenitors) and GFAP^+^/nestin^+^ B2/activated B1 cells. Cell nuclei were counterstained with DAPI (blue). (**b**) Quantitative immunohistochemistry revealed no difference in the total number of proliferating, i.e., BrdU-incorporating, cells between wild-type and m[A30P]h α synuclein mice. (**c**) In addition, we analyzed the relative proportion of the proliferating cell types in the V–SVZ and found a significant reduction in the population of B2/activated B1 neural stem cells. In addition, the proportion of marker-negative proliferating cells (nestin^−^GFAP^−^BrdU^+^) was significantly increased in mutant animals. The proportion of downstream progenitor cells, i.e., type A and C cells, was not significantly altered. Scale bar = 50 µm. All data are presented as mean ± standard error of the mean (S.E.M.); *n* = 4 each. Abbreviations: LV—lateral ventricle; WT—wild-type; BrdU—bromodeoxyuridine; GFAP—glial fibrillary acidic protein; Alpha4—Leu´9Ser-α4* nAChR animals.

Initially, we assessed both the proliferative activity as indicated by BrdU uptake and the relative distribution of the various neurogenic cell types within total numbers of proliferating cells after a short-term BrdU-labelling over 3 days prior to sacrifice. Neither Thy1-m[A30P]h α-synuclein mice nor Leu9′Ser hypersensitive α4* nAChR animals displayed alterations of BrdU-labelled proliferating cells in the V–SVZ (Figure 1a,b and Figure 2a,b). Interestingly, the proportion of B2/activated B1 cells—the latter representing the bona fide neural stem cells in this region—of all proliferating cells was significantly decreased in Leu9′Ser hypersensitive α4* nAChR mice. In Thy1-m[A30P]h α-synuclein animals the proportion of B2/activated B1 cells compared to the total amount of proliferating cells was also reduced, albeit not significantly (Figure 1c and Figure 2c). In addition, we found increased numbers of nestin and GFAP-negative proliferating (BrdU^+^) cells compared to controls (Figure 1c and Figure 2c). 

### 3.3. No Induction of Mid/Hindbrain Neurogenic Activity

We used the same animals as for the investigation of V–SVZ neurogenesis to assess whether dopaminergic neurodegeneration in the substantia nigra can act as a stimulus for the activation of local neural stem cells. The extent of dopaminergic neurodegeneration in Leu9′Ser α4* nAChR mice was already published elsewhere [32]; in Thy1-m[A30P]h α-synuclein mice, we analyzed the amount of striatal dopaminergic denervation as a measure of sufficient dopaminergic dysfunction, thus demonstrating a significant reduction in dopaminergic neurotransmission in our cohort (data not shown). As we and others already demonstrated, resident aNSCs can be detected in the PVRs of the entire ventricular system [16,35], though these are quiescent in vivo under physiological conditions. Whether local chronic neurodegeneration beyond an acute toxic impact in toxic PD animal models (such as 6-OHDA- or MPTP-induced animal models) might induce endogenous regeneration through activation of these aNSCs has not been studied so far.

**Figure 3:**
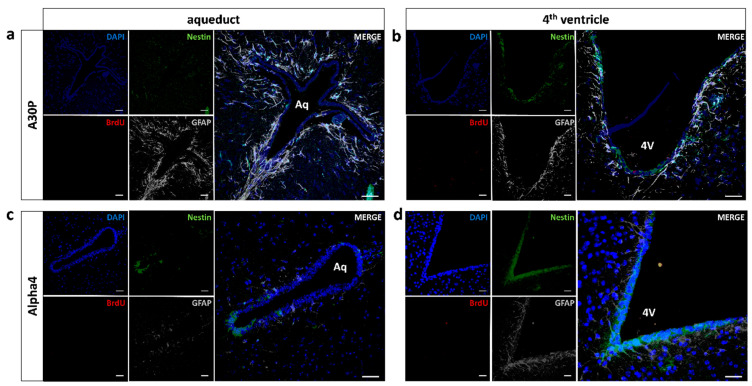
Proliferative activity in mid/hindbrain PVRs. Representative fluorescence image of mid/hindbrain periventricular regions. Daily BrdU injections over 3 days were performed prior to perfusion to label proliferating cells (red). To differentiate between the various cell types within a putative neurogenic cascade, quadruple immunostainings with DAPI/BrdU/nestin/GFAP were performed. Representative images of (**a**) the aqueduct and (**b**) the 4th ventricle in an h[A30P]m α-synuclein mutant mouse without evidence of a single BrdU^+^ cell (red). Further immunostainings were made for nestin (green) and GFAP (grey); cell nuclei were counterstained with DAPI (blue). Identical immunostainings were performed in (**c**) the aqueduct and (**d**) the 4th ventricle of L’S α4* nAChR mice. Scale bar = 50 µm. Abbreviations: Aq—aqueduct; 4V—4th ventricle; WT—wild-type; m[A30P]h—mutant human A30P α-synuclein mutant mice; BrdU—bromodeoxyuridine; GFAP—glial fibrillary acidic protein. All data are presented as mean ± standard error of the mean (SEM); *n* = 3 each (**a**;**b**); *n* = 4 (**c**;**d**).

We were not able to detect relevant numbers of proliferating, i.e., BrdU-incorporating, cells in the PVRs of the aqueduct and the fourth ventricle in wild-type animals, thus confirming literature findings [18]. In m[A30P]h α-synuclein mice, we detected very few BrdU^+^ cells in the PVR of the fourth ventricle, which were not embedded in a “structured” PVR comparable with the V–SVZ [16]. Surrounding the aqueduct, not a single BrdU^+^ cell could be detected in our study. Approximately half of the BrdU^+^ cells in the vicinity of the fourth ventricle were negative for neural precursors and mature astroglial cell markers. In absolute numbers of BrdU^+^ neurogenic subtypes, we found one nestin^+^/GFAP^−^ cell, three nestin^+^/GFAP^+^ cells and five nestin^−^/GFAP^+^ cells in the sum of all sections and animals (Figure 3a,b).

Regarding the second disease model, Leu9′Ser α4* nAChR mouse, the yield of proliferating cells within the mid/hindbrain was even lower, without a single BrdU^+^ proliferating cell in the respective PVRs of both the aqueduct and the fourth ventricle. We only detected two single BrdU^+^ cells in wild-type controls, though these cells were nestin^−^/GFAP^−^ (Figure 3c,d).

## 4. Discussion

Adult neurogenesis outlying the established neurogenic regions of the V–SVZ, the dentate gyrus and the hypothalamus has been discussed controversially for quite some time; while some studies detected relevant neurogenic activity in various brain regions or even a continuous turnover of midbrain dopaminergic cells in vivo [15], others found either minimal numbers of proliferating neuroprogenitor cells or mere gliogenesis [36,40,41,42]. Here, we assessed neurogenic activity in both the well-established neurogenic region of the V–SVZ and the PVRs in the proximity of the substantia nigra, namely mid/hindbrain PVRs, in two mechanistically different genetic animal models of PD.

The present study indicates only minor influences of the chronic dopaminergic denervation as it occurs in these two animal models on V–SVZ neurogenesis; indeed, overall proliferation was not altered in both phenotypes, and only L’S hypersensitive α4* nAChR mice present with a significant relative reduction of aNSCs in the V–SVZ. Nevertheless, similar results could be obtained in the m[A30P]h mouse, though they did not reach significance, most likely due to the small animal numbers in the present study. In both models, this finding does not translate into differences in overall numbers of proliferating cells indicated by BrdU uptake, either due to a compensation of reduced stem cell activity in downstream proliferation or due to our short-term labelling protocol. Regarding the L’S hypersensitive α4* nAChR mice, the minor effects might be due to the use of a strain with only ~30–40% nigral dopaminergic cell loss [31], which might not result in a sufficient dopaminergic deficit in the V–SVZ to cause major alterations in aNSC properties. Concerning the m[A30P]h α-synuclein mice, there are already a few studies on V–SVZ/olfactory bulb (OB) neurogenesis in the literature with overall reduction of the various subtypes of newly generated neurons in the OB, but with conflicting results on V–SVZ aNSC proliferation [30,43]. Since we applied our BrdU-labelling immediately prior to perfusion, our study was mainly designed to assess V–SVZ proliferation and not OB neurogenesis. The above-mentioned divergence, especially in V–SVZ-aNSC behavior, might be attributable to the different animal models in these studies, since m[A30P]h α-synuclein expression was either driven by specific promoters—CaMKII promotor in the study by Marxreiter et al. [30], Thy1 promotor in our model—or comprised a bacterial artificial chromosome (BAC) construct [43], which might influence both expression patterns and overall intracerebral α-synuclein load. Furthermore, the study of additional marker proteins to distinguish more precisely between different proliferative cell types within the V–SVZ could have provided additional information.

Our main focus here was to assess the putative activation of quiescent endogenous aNSCs in mid- or hindbrain PVRs when submitted to local PD-associated dopaminergic neurodegeneration. The presence of aNSCs in caudal PVRs after in vitro cultivation and their dopaminergic differentiation potential has been reported before [15,37], though in the majority of studies without signs of active postnatal neurogenesis under physiological conditions in vivo [16,35,44] and after exogenous growth factor stimulation [45]. Whether local degeneration of dopaminergic cells is sufficient to cause an arousal of resident aNSCs to overcome suppressive microenvironmental cues remains enigmatic. In the present study, we were not able to detect any significant proliferation in mid/hindbrain PVRs in our genetic PD models, which reproduce certain important neuropathological features of the human disease such as α-synuclein accumulation or progressive neurodegeneration [29,32].

There are of course certain limitations in our study, particularly regarding small animal numbers and the short-term protocol over three days of BrdU application, which might not have detected very slowly proliferating cells [44]. On the other hand, such minimal proliferation might in any case not account for the extensive dopaminergic cell loss seen in the human disease. In addition, we were only recently able to demonstrate that the inhibition of proliferation in caudal PVRs is mainly mediated by norepinephrinergic (NErgic) innervation [17], indicating that dopamine might not play a substantial role in aNSC regulation in these caudal regions. Of note, these innervation patterns are already established during late embryonic development [46]. In line with that, growth factor infusions such as EGF/FGF-2 in these regions were only able to activate aNSC proliferation and gliogenesis but not neurogenesis in the mid/hindbrain regions [45], arguing for a strong local neurogenesis restrictive cue. Only inhibiting norepinephrinergic innervation so far was able to overcome this barrier and induce local neurogenesis in these regions [17]. Therefore, dopaminergic denervation in regions with minimal dopaminergic input under physiological conditions might not exert any relevant influence. While we did not measure NE content in mid/hindbrain regions in the current study, it has been reported that m[A30P]h-α-synuclein [39] mice possess normal NE content in forebrain regions at the age of 8 months, and Leu9´Ser hypersensitive α4* nAChR receptor mice have unchanged NE neurons in the locus coeruleus at the same age [32]. Thus, even though not measured, our data indicate an absence of changes in NE content at least in the hindbrain PVRs. Regarding the animal models, we chose two mechanistically relevant genetic models with significant dopaminergic degeneration, displaying two neuropathological hallmarks of PD: α-synuclein-containing inclusion bodies, and a chronic–progressive dopaminergic degeneration [29,31]. However, we cannot exclude that our findings might differ in other genetic PD models and PD patients themselves, especially if these might more affect the NE system (for a review, see [47]).

## 5. Conclusions

Although the existence of quiescent neural stem or progenitor cells in vivo in mid/hindbrain periventricular regions with dopaminergic differentiation potential in vitro has been demonstrated before, the dopaminergic deficits in these two genetic PD animal models do not induce endogenous regeneration through proliferation of resident neural stem or progenitor cells adjacent to the sites of dopaminergic pathology, the midbrain periventricular regions. This might possibly arise from a lack of physiological dopaminergic signaling in these regions. In addition, while the dopaminergic deficit in these animals did not lead to a decrease of total proliferating cells, it did reduce B2/activated B1 cells in the ventricular–subventricular zone of the lateral ventricles.

## Data Availability

The raw data supporting the conclusions of this article will be made available by the authors, without undue reservation.
